# LC-MS/MS and GC/MS Profiling of *Petroselinum sativum* Hoffm. and Its Topical Application on Burn Wound Healing and Related Analgesic Potential in Rats

**DOI:** 10.3390/metabo13020260

**Published:** 2023-02-11

**Authors:** Meryem Slighoua, Ismail Mahdi, Fatima Zahrae Moussaid, Omkulthom Al Kamaly, Fatima Ez-zahra Amrati, Raffaele Conte, Aziz Drioiche, Asmaa Saleh, Abdelilah Iraqi Housseini, Amina Bari, Dalila Bousta

**Affiliations:** 1 Laboratory of Biotechnology, Environment, Agro-Food, and Health (LBEAS), Faculty of Sciences, University Sidi-Mohamed-Ben-Abdellah (USMBA), Fez 30050, Morocco; 2 AgroBioSciences Research Program, Mohammed VI Polytechnic University, Lot 660-Hay Moulay Rachid, 11, Ben-Guerir 43150, Morocco; 3 Department of Pharmaceutical Sciences, College of Pharmacy, Princess Nourah bint Abdulrahman University, P.O. Box 84428, Riyadh 11671, Saudi Arabia; 4 Research Institute on Terrestrial Ecosystems (IRET)—CNR, Via Pietro Castellino 111, 80131 Naples, Italy; 5 Laboratory of Innovative Materials and Biotechnology of Natural Resources, Faculty of Sciences, Moulay 19 Ismail University, Meknes 50000, Morocco

**Keywords:** *Petroselinum sativum* Hoffm., LC-MS/MS, GC-MS, analgesic activity, burn wound healing

## Abstract

Parsley (*Petroselinum sativum* Hoffm.) is renowned for its ethnomedicinal uses including managing pain, wound, and dermal diseases. We previously highlighted the estrogenic and anti-inflammatory properties of parsley and profiled the phytochemistry of its polyphenolic fraction using HPLC-DAD. To extend our investigation, we here characterized the phytochemical composition of the hydro-ethanolic extract using LC-MS/MS and GC-MS upon silylation, and evaluated the antioxidant, analgesic, antimicrobial, and wound healing activities of its hydro-ethanolic and polyphenolic fraction. The antioxidant property was assessed using FRAP, DPPH, and TAC assays. The antimicrobial activity was tested against four wound infectious microbes (*Staphylococcus aureus*, *Pseudomonas aeruginosa* and *Candida albicans*). The analgesic effect was studied using acetic acid (counting the number of writhes) and formalin (recording the licking and biting times) injections while the wound healing activity was evaluated using burn model in vivo. The LC-MS/MS showed that the hydro-ethanolic contains four polyphenols (oleuropein, arbutin, myricetin, and naringin) while GC-MS revealed that it contains 20 compounds including malic acid, D-glucose, and galactofuranoside. The hydro-ethanolic (1000 mg/kg) decreased abdominal writhes (38.96%) and licking time (37.34%). It also elicited a strong antioxidant activity using DPPH method (IC_50_ = 19.38 ± 0.15 µg/mL). Polyphenols exhibited a good antimicrobial effect (MIC = 3.125–12.5 mg/mL). Moreover, both extracts showed high wound contraction by 97.17% and 94.98%, respectively. This study provides evidence that *P. sativum* could serve as a source of bio-compounds exhibiting analgesic effect and their promising application in mitigating ROS-related disorders, impeding wound infections, and enhancing burn healing.

## 1. Introduction

Pain and inflammation are strongly involved in the healing process of the wound. Therefore, conventional medicines are often used to mitigate the intensity of pain and inflammation [1]. After an injury, the skin regenerates through the process of wound healing. The interaction between several cellular elements (fibroblasts, keratinocytes, endothelial cells, and macrophages/monocytes), and the constituents of the extracellular environment (collagen and fibronectin) during the wound healing phases, promote the wound contraction, and restore tissue integrity [2]. The wound healing provides the ideal micro-environment at the injured surface to achieve the maximum skin repair [3]. However, several disorders may affect the ability of healing such as mechanical stresses, toxic agents, or infections. In most situations, the molecular events that accelerate the nociception response are similar whether the pain is of extrinsic or intrinsic origin [2].

By reacting with biological components such as proteins and nucleic acids, excessive levels of reactive oxygen species (ROS) disrupt intrinsic tissue disability and lead to loss of function [4,5]. For instance, high concentrations of H_2_O_2_ can cause oxidative damage and thus delay healing, while low concentrations can act as a signaling molecule and promotes healing. However, all the advancements achieved against oxidative stress, the production of ROS during injuries, and their impact on the healing process still constitute a major health challenge [6].

Parsley, whose scientific name is *Petroselinum sativum* Hoffm., is a plant belonging to the Apiaceae family. Currently, it is cultivated all over the world and has been used as food, cosmetic ingredient, and for pharmaceutical purposes [7]. Parsley has several biological and pharmacological activities, mainly spasmolytic, antioxidant, immunomodulating, gastrointestinal, and antidiabetic attributes [8]. These various virtues are due to its bioactive phytoconstituents such as carotenoids, flavonoids, coumarin, and vitamins [9].

Several previous investigations have stated that herbal and plant-based ingredients activate the wound and cutaneous healing process. These include many medicinal and aromatic plants (MAP) such as turmeric (*Curcuma longa*), centella (*Centella asiatica*), tree peony (*Paeonia suffruticosa*), and aloe vera (*Aloe barbadensis*) [10,11]. Additionally, in traditional medicine, *Petroselinum* species including *P. sativum* were reported to be used in Anatolia, Turkey for wound healing purposes [12] as well as in treating some dermal diseases [13,14]. However, very limited studies were devoted to providing scientifically sound data to test this claim. Very recently, Thangavelu et al. (2022) showed that the leaf methanolic extracts of *P. crispum*, whose synonym is *P. sativum*, exhibited potent wound healing and anti-inflammatory activities on the human lung cancer cell lines by enhancing cell migration [15]. Interestingly, in another study, parsley was used as a maintenance diet for cutaneous closure of wounds in rabbits as a postoperative care after the surgery [16]. Elsewhere, *P. crispum* was also investigated for immunomodulatory and wound healing activities [17]. Moreover, the analgesic use of parsley in folklore medicine was also reported and demonstrated in vivo using seeds hydroalcoholic extract [14,18]. Other studies corroborated the same findings [19,20].

The growing antimicrobial resistance of microbes responsible for skin infections blew up the research on the potential of MAP preparations in antimicrobial therapeutics. In fact, many phytochemicals are shown to be effective against microbial infections [21,22]. In wounds, the skin barrier is breached and becomes susceptible to microbial infections by bacteria, fungi, and/or viruses, that delay the healing process [23]. These pathogens include Gram-positive bacteria such as *Staphylococcus aureus* and *Streptococcus pyogenes*, and Gram-negative bacteria including *Escherichia coli*, *Pseudomonas aeruginosa*, and *Klebsiella species*, and fungi—mainly *Candida* and *Aspergillus* [24]. Hence, minimizing the predisposition of wounds to infections is required in wound surveillance to reduce the infection rate [25].

We previously explored the estrogenic as well as the anti-inflammatory activities of *P. sativum* Hoffm. in vivo and characterized the chemical composition of its polyphenolic fraction [26]. Recognizing the promising therapeutic potential of this plant species and to follow up on our previous findings, we conducted this investigation that aimed to (1) annotate the phytoconstituents of the hydro-ethanolic extract of *P. sativum* Hoffm. using both LC-MS/MS and GC-MS to identify the maximum number of phytocompounds and (2) monitor the analgesic, antioxidant, antimicrobial, and burn healing effects of both the hydro-ethanolic extract and polyphenolic fraction of the plant. This study is the first to demonstrate the pharmacological relevance of parsley as a source of wound healing and analgesic biochemicals.

## 2. Materials and Methods

### 2.1. Plant Material

Aerial parts of *P. sativum* Hoffm. were collected before sunrise in the Taounate region (north of Morocco). The botanical name was checked by Pr. Bari Amina, at the laboratory of Biotechnology, Environment, Agro-Food, and Health, at Sidi Mohamed Ben Abdellah University. A sample was deposited under a voucher number (18TA5001) at the herbarium of the Faculty of Sciences-Fez.

### 2.2. Animal Material

In this study, we used Swiss albino mice rearing in an animal house that has a relative humidity of 50 to 55%, with an average temperature of 22 ± 2 °C, and a day/night cycle of about 12/12 h. Animals were given free access to water and food. The average weight of mice used was 30 ± 4 g. The handling and manipulation of animals were up to the standards of the directive EEC/86/EEC of the European community [26,27]. The experiment was approved by the institutional ethical committee of the Faculty of Sciences Dhar El Mehrez, Sidi Mohamed Ben Abdallah Fez University, Morocco (#04/2019/LBEAS).

### 2.3. Preparation of the Hydro-Ethanolic Extract

Aerial parts were left to the laboratory to dry for one week, then grinded using a blender. The grind (30 g) was macerated in 210 mL of ethanol 70% for three days [26]. The macerate was filtered, evaporated, and dried (rotary evaporator, 37 °C) [26,28]. 

### 2.4. Preparation of the Polyphenolic Fraction 

One hundred grams of well ground *P. sativum* powder was subjected to three extractions in methanol (300 mL × 3) at 50 °C for 3 h. Subsequently, solvent was evaporated, and the obtained extract was dissolved in distilled water (500 mL) and extracted in hexane (200 mL × 3) and then in chloroform (200 mL × 3) to remove caffeine and chlorophyll residues. Next, the aqueous phase was extracted in ethyl acetate (200 mL × 3) which was evaporated later. Using 300 mL of distilled water, the residue was dissolved and then lyophilized [29,30].

### 2.5. Phytochemical Analysis by LC-MS/MS

The chemical profiling of the hydro-ethanolic extract of *P. sativum* was performed using ultra-high performance liquid chromatography (Shimadzu, Nexera XR LC 40) coupled with mass spectrometry (LCMS 8060, Shimadzu Italy, Milan, Italy). The heating and nebulization gas flow was set to 10 and 2.9 L/min, respectively. The drying gas flow was at 10 L/min, the DL temperature was at 250 °C, the heating block temperature was 400 °C, and the interface temperature was 300 °C. Separation of compounds was carried out using C18 column, 3 × 100 mm, 2.6 μm (Phenomenex, Torrance, CA, USA). The mobile phase consisted of acetonitrile (A) and water containing 0.01% formic acid (B). The extract was added to acetonitrile and water (1:1) and then diluted (1/50) in acetonitrile and injected [31]. The ion currents’ acquisition was carried out in single ion monitoring (MRM) mode in negative ESI ionization. The analyzed molecular adducts were, respectively, 579, 539, 317, and 271.2 for Naringin, Oleuropein, Myricetin, and Arbutin.

### 2.6. Phytochemical Analysis by GC-MS

Phytochemical identification of the hydro-ethanolic extract of *P. sativum* was carried out using GC-MS after silylation. This latter is based on dissolving 1 mg of the grind in 100 mL of HMDS-TMCS-Pyridine 3:1:9 (*v*/*v*/*v*) reagent. After 30 min incubation [32], the extract was injected into the GC-MS apparatus (Agilent Technologies MASS Selective Detector, 5973 Network) with a capillary column Agilent 19091S-433 model, 30 m in nominal length—0.25 mm in diameter and 0.25 μm thick. Helium served as the carrier gas, and the total flow of 31.4 mL/min and a split ratio of 30:1 was used. The temperature program was between 60 and 300 °C and maintained for 20 min of the run time. The detector temperature was set to 260 °C. Splitless injection was used [33].

### 2.7. Determination of Total Phenol and Flavonoid Contents

The quantification of total phenol content (TPC) and total flavonoid content (TFC) was carried out calorimetrically using the methods described by Slinkard and Singleton (1977) [34]. TPC content was expressed in milligrams (mg) of gallic acid equivalents per gram (g) of dry weight of extract (mg GAE/g DW) while the values of TFC were expressed in mg of quercetin equivalent per g of dry extract (mg EQ/g DW).

### 2.8. Assessment of Antioxidant Activity

#### 2.8.1. Scavenging of the Free Radical (DPPH)

In this study, the DPPH test performed by Brand-Williams in 1995 [35] was followed. Methanol (100 μL) was mixed with 750 μL of DPPH solution, incubated for 30 min, and then absorbance was measured at OD_517nm_. BHT (butylated hydroxytoluene) was used as the standard antioxidant. To calculate the percentage of inhibition (IP) of DPPH, the following formula was used:

$$\mathrm{IP}\;\left(\%\right)=\left(A0\;–\frac{A}{A0}\right)\times 100$$

IP: Inhibition Percentage.
A0: OD of DPPH solution in the absence of the extract (negative control).
A: OD of DPPH solution containing the extract.

#### 2.8.2. Ferric Reducing Antioxidant Power (FRAP)

The FRAP assay was performed according to the method described by Oyazu (1986) [36]. Briefly, 200 µL of each extract was mixed with the buffer solution (0.5 mL) (0.2 M, pH = 6.6) and potassium ferricyanide [K_3_Fe (CN)_6_] at 1% (0.5 mL). The solution was kept at 50 °C in a water bath for 20 min. Next, to acidify to solution, 500 µL of trichloracetic acid at 10% was added to the solution and centrifuged for 10 min at 3000 rpm. Five hundred microliter of the top layer of the solution was mixed with distilled water (500 µL) and FeCl_3_ 0.1% (100 µL). Ascorbic acid was used as standard, and the absorbance was read at OD_700nm_. The values were expressed as EC_50_ (mg/mL). The EC_50_ was calculated using the standard curve.

#### 2.8.3. Total Antioxidant Capacity Test (TAC)

The TAC was evaluated by mixing 25 µL of every studied extract with 1 mL of liquid reactive solution (28 mM Na_3_PO_4_, 0.6 M H_2_SO_4_, and 4 mM (NH_4_)_2_MoO_4_). Following incubation (90 min, 95 °C), the absorbance values were read at OD_695nm_. The antioxidant potential was determined in mg of equivalent of ascorbic acid per gram of extracts (mg EAA/g of extracts) [37,38].

### 2.9. Antimicrobial Activity

The extracts were evaluated for their potential antimicrobial effect against three human pathogenic strains including one Gram-negative bacterium (*Pseudomonas aeruginosa* CECT118), one Gram-positive bacterium (*Staphylococcus aureus* CECT976), and one fungal species (*Candida albicans* ATCC 10231). The antimicrobial susceptibility of the three strains was evaluated using agar well diffusion and broth dilution methods. 

#### 2.9.1. Agar Well Diffusion Assay

The method of diffusion in agar wells was used to carry out the qualitative test, a widely-known assay to check the antimicrobial activity of herbal extracts [39,40]. From fresh overnight cultures, microbial suspensions were prepared and adjusted to 0.5 McFarland corresponding to 10^6^ CFU/mL [41,42]. Afterwards, 5 mL of soft agar (agar 4 g/L) inoculated with 100 µL of the microbial suspension (10^6^ CFU/mL) of each strain were poured over the surface of each plate. After solidification, wells of 8 mm diameter were aseptically punched using glass Pasteur pipets. Then, 50 μL of each extract was dissolved in the appropriate solvent (40 mg/mL) and introduced into the appropriate wells [43]. Under the same conditions, the controls were established using the solvent only. The Petri dishes were then incubated at 37 ± 2 °C for 48 h. The extracts diffuse through the agar medium and the formation of inhibition zones surrounding the wells indicate positive antimicrobial activities. Streptomycin (1 mg/mL) and fluconazole (5 mg/mL) served as positive controls for bacteria and fungi, respectively.

#### 2.9.2. Determination of the Minimum Inhibitory Concentration (MIC)

The MIC of the hydro-ethanolic and polyphenolic extract was checked using microdilution assays according to the standards of the NCCLS [44] in 96-well microtiter microplates. First, 50 μL of the culture broth was introduced into each well of the microplates. Next, seven concentrations of the hydro-ethanolic extract and the polyphenolic fraction (0.78–50 mg/mL), streptomycin and fluconazole (0.078–5 mg/mL) were prepared in both LB and YPG in sterile haemolysis tubes. Each microplate well was inoculated by 100 µL of LB liquid culture medium for bacteria and YPG liquid culture medium for yeast strains, 50 µL of each extract, and then concentrations were carried out by successive two-fold dilutions. Afterwards, 50 μL of the microbial suspensions, whose turbidity was checked in the same way as described above, were inoculated into the microplate’s wells. The microplates were incubated at 37 °C for the bacterial strains and at 30 °C for *Candida albicans* ATCC 10231 under 150 agitation rpm for 24 h. By the end of the incubation, we added 20 µL of 2,3,5-triphenyltetrazolium chloride into each plate’s well and incubated the plate for 2 h. The formation of a pinkish coloration indicates that the growth is due to the activity of the dehydrogenases. The MIC corresponds to the lowest concentration that does not produce a red colour [45].

### 2.10. Analgesic Activity In Vivo

#### 2.10.1. Abdominal Writhes

Five groups containing five mice each (25 mice) were prepared. The control mice were given 10 mL/kg of NaCl 0.9% and Tramadol (10 mg/kg) was used as a standard (reference drug) [46]. The other groups received two doses of the hydro-ethanolic extract (500–1000 mg/kg) and one dose from the polyphenolic fraction (200 mg/kg). One hour later, 1% acetic acid was injected by intraperitoneal route at 10 mL/kg rate. Ten minutes later, the number of writhes were determined over a duration of 20 min [47,48].

#### 2.10.2. Formalin Induced Pain

First, we injected 10% formalin (20 mL) into the right posterior paw of each animal. With the help of a stopwatch, we recorded the licking and biting times. The initial nociceptive response is the sum of the seconds passed in licking and biting from 0 to 5 min after the injection of formalin while the second phase was from 15 to 30 min [49,50]. Half an hour beforehand, the animals were subjected to oral prior treatment by the test extract, NaCl or Tramadol [51].

### 2.11. Wound Healing Activity In Vivo

#### 2.11.1. Ointments Preparation

The preparation of the ointment was carried out at 10% (*w*/*w*) by adding 1 g of the extract to 9 g of Vaseline, and melted using a bain-marie at a temperature of 50 °C. After homogenization, the preparations were kept at 4 °C in sealed containers [4].

#### 2.11.2. Induction of Burn Injuries

In this experiment, 4 groups containing 5 rats each were prepared. The first group represents the negative control (Vaseline), the second group represents the positive control (Madecassol (1%)), and the third and fourth groups represent the groups treated with the hydro-ethanolic and polyphenolic extracts of *P. sativum* Hoffm., respectively. According to the protocol of Heidari et al., (2019), after anesthesia of rats with pentobarbital (50 mg/kg) and shaving the dorsal part with an electric clipper [52], the induction of the burn was carried out on the shaved part using an aluminum rod (1.7 cm) heated to 110 °C for 10 s. After 24 h, the treatment was started by applying the ointments to the burned zones for 25 days while photographing the healing progress using a digital camera and the ruler used as a scale. *ImageJ*^®^ software was used to analyze the images and measure the rate of wound contraction using the following formula [53]:

$$WC\;\left(\%\right)=\left[\frac{\left(WS0-WSSD\right)}{WS0}\right]\times 100$$

*WC* (%) = Rate of wound contraction.
*WS*0 = Size of the wound at the first day.
*WSSD* = Size of the wound at each specific day.

### 2.12. Statistical Analysis

Results obtained from each experiment were treated by using a one-way ANOVA followed by the post-hoc analysis with Tukey’s test in GraphPad Prism 6 software. Values were expressed as mean ± SD and the significance level was set at “*p* < 0.05”. The significant differences between treatments were shown using different superscript letters (a, b, c, etc.).

## 3. Results

### 3.1. Phytochemical Analysis by LC-MS/MS 

**Figure 1:**
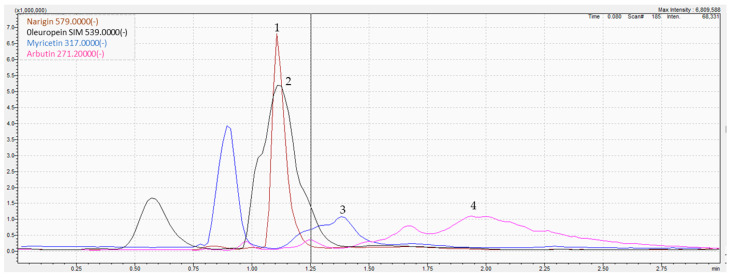
LC-MS/MS chromatogram of the hydro-ethanolic extract of *P. sativum.* (**1**) Naringin, (**2**) Oleuropein, (**3**) Myricetin, (**4**) Arbutin.

The determination of parsley phytoconstituents was performed according to the molecular weight of the fragments generated. The analysis of the hydro-ethanolic extract revealed the presence of four molecules namely oleuropein, arbutin, myricetin, and naringin, all classified as polyphenols (Figure 1 and Table 1). For instance, negative ion ESI-MS/MS spectra of oleuropein resulted in the formation of *m/z* 539, a pseudomolecular ion, as the sole base peak of the ESI-MS spectra, while the MS/MS products were abundant (e.g., 307 and 275). Similarly, myricetin (*m/z* 317) was fragmented to four main products (150.8, 178.8, 270.9, and 286.9). The MS/MS spectra of identified compounds, annotations, and their characteristic fragmentation patterns are presented in Table 1. XIC chromatograms of identified compounds are provided in the Supplementary Material S1.

### 3.2. Phytochemical Analysis by GC-MS

Silylation is the appropriate method to check for thermolabile and non-volatile phytochemicals by GC-MS. It consists of replacing the active hydrogen in =NH, –NH_2_, –OH, –COOH, or –SH by a trimethylsilyl group. This showed that the hydro-ethanolic of *P. sativum* contained twenty compounds with a total of 99.9%. According to the area percentage, the most dominant compounds were malic acid (13.52%), D-glucose (13.529%), D-mannitol (10.957%), and talose (9.758%) (Table 2). 

### 3.3. Estimation of Total Phenol and Flavonoid Contents

The total phenols and flavonoids contents contained in the hydro-ethanolic extract of *P. sativum* were 34.55 ± 3.74 mg GAE/g of extract and 16.46 ± 0.06 mg QE/g of extract, respectively. 

### 3.4. Antioxidant Activity

#### 3.4.1. DPPH and FRAP Assays

**Figure 2:**
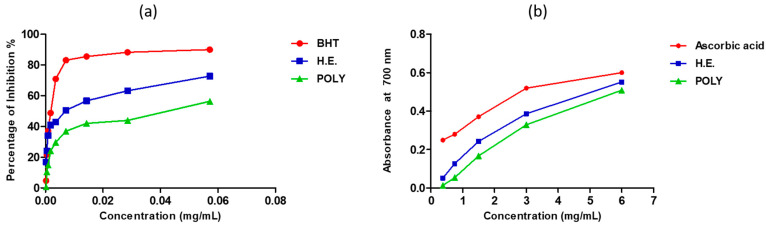
Antioxidant activity of the hydro-ethanolic and polyphenols extracts of *P. sativum* Hoffm. using DPPH (**a**) and FRAP (**b**) assays.

The antioxidant activity of our extracts was tested by DPPH and FRAP tests. The inhibition of the free radical DPPH by the hydro-ethanolic was greater than that of the polyphenols (Figure 2a). The 50% inhibition concentration (IC_50_) of the hydro-ethanolic extract was seen at 19.38 ± 0.15 µg/mL, and that of the polyphenols was obtained at 40.36 ± 1.47 µg/mL (Table 3). However, these results are significantly lower than those obtained using butylated hydroxytoluene (BHT) (IC_50_ = 1.97 ± 0.1 µg/mL) (Figure 2a and Table 3). The ferric reducing power of our extracts showed that hydro-ethanolic extract was more effective than polyphenols, but the potential of both extracts was slightly lower than that of the standard antioxidant ascorbic acid (Figure 2b & Table 3). 

#### 3.4.2. Total Antioxidant Capacity (TAC)

TAC assay showed that the hydro-ethanolic has a greater total antioxidant capacity (175.2 ± 6.360 mg EAA/g) comparing to polyphenols (148.2 ± 13.86 mg EAA/g).

### 3.5. Antimicrobial Activity

The antimicrobial assay on the plate showed that the hydro-ethanolic extract elicited an antibacterial and anti-fungal activity against *P. aeruginosa* and *C. albicans*, respectively, while *S. aureus* was showed to be resistant. In contrast, the polyphenolic fraction exhibited a higher inhibition zone diameter against the three tested pathogens with a noticeable inhibitory effect toward *P. aeruginosa* (Table 4). Nevertheless, positive controls, streptomycin (1 mg/mL) and fluconazole (5 mg/mL), were relatively more potent comparatively to the tested extracts.

The antimicrobial effects against the three species were noticed at MIC values ranging from 3.125 to 12.5 mg/mL. The polyphenols were the most active as they inhibited the three species with a prominent effect towards *S. aureus* (MIC = 3.125 mg/mL). Worth noting is that the broth dilution assay corroborated the non-toxic effect of the hydro-ethanolic extract against *S. aureus* even at the highest concentration tested, 50 mg/mL (Table 5).

### 3.6. Analgesic Activity

#### 3.6.1. Abdominal Writhes

**Figure 3:**
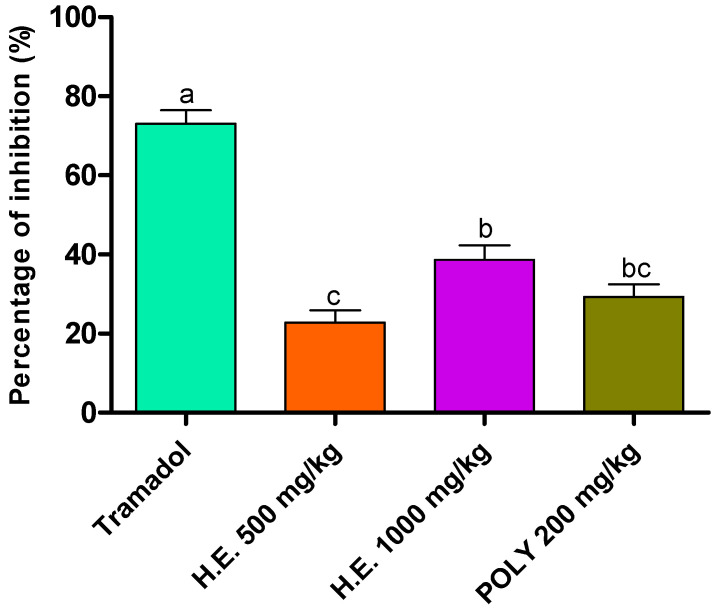
Inhibitory effect of the hydro-ethanolic (H.E.) and polyphenolic (Ploy) extracts of *P. sativum* Hoffm. and Tramadol (positive control) on contortions in mice. The different letters in superscript (a, b, c) indicate the significant difference between treatments at *p* < 0.05.

Analgesic activity was evaluated using acetic acid method. Although less efficient than the standard drug (Tramadol), the hydro-ethanolic (1000 mg/kg) and polyphenols (200 mg/kg) induced a significant decrease in the number of writes by 38.96 and 29.23%, respectively, followed by the hydro-ethanolic extract at 500 mg/kg by 23.07% as compared to the negative control animals which we have taken as reference to calculate the percent of inhibition (Table 6 and Figure 3).

#### 3.6.2. Formalin Induced Pain

**Figure 4:**
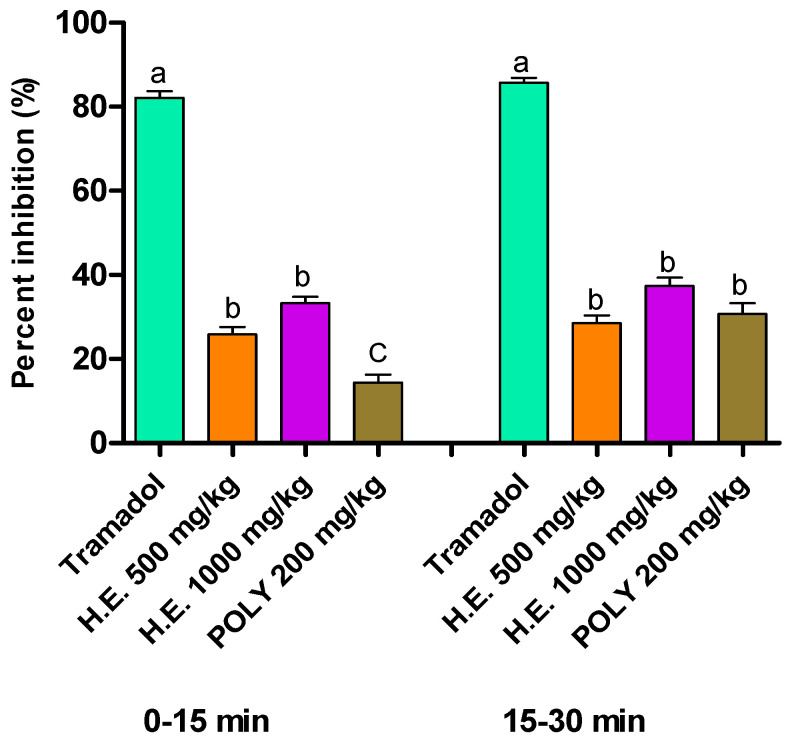
Percent of inhibition of formalin-induced pain in mice following the application of hydro-ethanolic (H.E.) and polyphenolic (Ploy) extracts of *P. sativum* Hoffm. and Tramadol (positive control). Inhibition rates were determined compared to the non-treated animals. The different letters in superscript (a, b, c) indicate the significant difference between treatments at *p* < 0.05.

In the formalin test, the hydro-ethanolic extract at the dose 500 and 1000 mg/kg of *P. sativum* Hoffm. elicited a significant reduction in response to nociception during the first phases (0–5 min) and the second phases at the doses 500 and 1000 mg/kg of the hydro-ethanolic extract and polyphenols comparatively to the control mice. Worth noting is that Tramadol was the most potent in both phases by up to 85.7% reduction in response time. During the first phase, hydro-ethanolic extract reduced the response time by 33.32% and 25.86% using 1000 and 500 mg/kg, respectively. In the second phase, both hydro-ethanolic and polyphenols extracts showed a significant reduction in response time by 28.55% using hydro-ethanolic at 500 mg/kg, 37.35% using hydro-ethanolic at 1000 mg/kg, and 30.76% using polyphenols at 200 mg/kg (Table 7 and Figure 4). 

### 3.7. Wound Healing Activity

**Figure 5:**
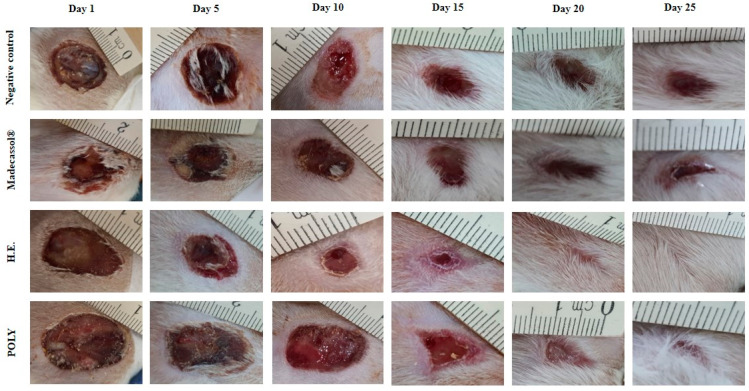
Morphological aspects of cutaneous burn healing process upon application of the hydro-ethanolic (H.E) and Poly (Polyphenols) extracts of *P. sativum* Hoffm. and the control groups during 25 days of treatment. The rate of the healing activities induced by each application were compared to each other. As shown in Figure 6, the hydro-ethanolic and polyphenols extracts elicited a high wound contraction at the 5th day by 46.45% and 38.7%, respectively. At the 25th day, the hydro-ethanolic extract elicited the highest healing (up to 97.17%), followed by the polyphenols and Madecassol ointment with almost the same percentage of inhibition (94.98% and 94.27%, respectively) while the negative control group was improved by only 80.62%.

The effects of the ointments prepared from hydro-ethanolic and polyphenols extracts are presented in Table 8. We showed that both applied extracts induced a significant healing activity as compared the untreated animals. Figure 5 illustrates the healing progression using the extracts and the controls from the 1st to the 25th day. The negative control group (Vaseline^®^), positive control group (Madecassol^®^), and polyphenols did not induce a complete wound closure. In contrast, the hydro-ethanolic extract induced a complete cicatrization of the wound at the 20th day.

## 4. Discussion

Parsley is a medicinal plant largely used a garnish and food-flavoring agent but also in traditional pharmacopoeia to treat several diseases such as inflammation, diabetes, cancer, digestive disorders, and kidney stones [14,54]. Nevertheless, many of these traditional applications remain to be discovered and proven. Here, we annotated the phytoconstituents of its hydro-ethanolic extract and studied the analgesic, antioxidant, and antimicrobial of the hydro-ethanolic and polyphenol extracts of parsley (*P. sativum* Hoffm.). Most importantly, we believe that this is the first study to assess the wound healing properties of *P. sativum* Hoffm. extracts using animal models.

The LC-MS/MS analysis of *P. sativum* corroborated the presence of some polyphenols known for their biological effects such as oleuropein (antioxidant, anti-atherogenic, antimicrobial, anti-cancer, antiviral activity, anti-inflammatory, hypoglycemic, and hypolipidemic activities) [55], arbutin (wound healing, anti-inflammatory, antioxidant, analgesic, anticancer, antiparkinsonic, and hypoglycemic effects) [56], myricetin (antimutagen, anti-ulcer, anticarcinogen, antioxidant, antibacterial, anti-diabetic, cardioprotective, anti-amyloidogenic, anti-inflammatory, and antiviral activities) [57,58], and naringin (anti-atherosclerosis, anti-diabetic, neuroprotective, cardioprotective, rheumatologic, and osteoporosis disorders). Moreover, the GC-MS analysis of the hydro-ethanolic extract of *P. sativum* Hoffm. showed the presence of some compounds renowned for their pharmacological activities like malic acid (anti-thrombotic) [59], D-glucose (anti-cancer, anti-diabetic) [60,61], carbazoles (antifungal) [62], and myo-inositol (against both male and female infertilities) [54]. The chemical compounds identified here are likely to be behind the biological and pharmacological activities demonstrated in this study. In line with other works, our results show the presence of phytochemicals in parsley such as alkaloids, polyphenols, and sugars that contribute to the structure of the flavonoid glycosides and amino acids [9,14,62].

The analgesic activity was tested by two protocols: abdominal writhes and formalin induced pain as described by Ganguly et al., 2016 [63]. Our findings suggest that the analgesic activity of *P. sativum* Hoffm. could be related to its effect on prostaglandin biosynthesis [64]. In fact, acetic acid induces the secretion of prostaglandins (PGE2 and PGE2α), partly involving peritoneal receptors and inflammatory discomfort [65]. Other studies showed the analgesic effect of the ethanolic extract of parsley prepared at 100, 150, and 200 mg/kg acts by decreasing both phases of pain using the formalin test [19]. Using this assay, there is two distinct biphasic nociceptive responses known as early and late phases [66]. Molecules that target precisely the central nervous system (CNS) can inhibit both phases by a similar mechanism. However, drugs acting on the peripheric nervous system (PNS) inhibit the late phases only [67,68]. The early phase could be triggered by the nociceptors induction in the paw reflecting the centrally mediated pain, while the late phase is activated by the release of pro-inflammatory agents such as serotonin, bradykinin, histamine, and prostaglandins [68,69]. However, this could be also due to the central nociceptive neuron’s activation [69,70].

Balance between the release of anti and pro-inflammatory cytokines and analgesic mediators induces the chronicity of pain [71,72]. In a recent study, we demonstrated the anti-inflammatory effect of the hydro-ethanolic and polyphenolic extracts from *P. sativum* Hoffm. [26]. As the healing process is strongly associated with the inflammatory response by the intervention phase of monocytes and neutrophils, the proliferation of epithelial cells and fibroblasts, the synthesis of collagen, and the action of keratinocytes fibroblasts [73]. We evaluated the potential healing activity of our extracts in rats and showed that application of ointments prepared from the hydro-ethanolic extract and polyphenolics induced a significant cicatrizing effect as compared the untreated animals. This is most likely due to the presence of bioactive compounds that support inflammation to repair lesions and accelerate cell regeneration in damaged tissues. In fact, several phytochemicals such as polysaccharides, alkaloids, and saponins have been demonstrated to have wound healing properties [74]. For instance, triterpenes isolated from *Centella asiatica* stimulated glycosaminoglycans synthesis and ameliorated collagen remodeling [75]. In addition, madecassoside from this plant administered orally improved both collagen synthesis and angiogenesis. Worth noting is that *Arctium lappa* L. was able to monitor adhesion of dermal fibroblasts and regulate their gene expression by targeting the Wnt/β-catenin signaling pathway which is well documented as a major wound regulator [76]. Other molecules including apigenin are known for controlling the wound healing process [77].

As nutrients are important factors in wound healing, many studies showed that nutrient shortage is likely linked to the delayed healing of wounds [78]. For instance, vitamin K is mandatory during the first phase (hemostasis). Its deficiency alters wound repair, hemorrhage, and infection [79]. Interestingly, parsley is known as one of the leafy green vegetables to be rich in vitamin K [78]. In addition, as malic acid is one of the most abundant molecules identified in this study, its action as a wound healing agent could corroborate a previous study that showed that purified fractions from the leaves of *Sempervivum tectorum* L. harboring high contents of malic acid promote cellular proliferation and migration [80,81]. Other studies have investigated the role of malic acid derived polymers on muscle regeneration and bone repair [82].

Flavonoid contents of *P. sativum* Hoffm. were different from those obtained by Pereira et al., (2014) [83] who showed a low TFC in the hydro-ethanolic extract of parsley. This can be explained by the difference in the extraction method used, geographical regions of growth, seasonal variations, harvesting time, and postharvest treatment [5]. Previous studies have shown similar results regarding the TFC of parsley (27.2 mg QE/g) [84,85]. Other works revealed many factors that can influence the content of TPC such as genetic and extrinsic factors namely climatic and geographic ones [86]. There is also the duration of storage, chemotype, and the degree of maturation of the plant which have a strong influence on the polyphenols contents [87].

Here, the antioxidant activity was monitored using DPPH, FRAP and TAC tests. The difference seen between the hydro-ethanolic extract and the polyphenolics could be related to the presence of other chemicals having antioxidant activity other than the polyphenols. As previously reported, this antioxidant ability can be due to the presence of malic acid in *P. sativum* Hoffm. [88,89]. Other studies have shown the significant antioxidant capacity of the aerial part of parsley due to the presence of flavonoid [90,91]. For instance, Marin et al. (2016) demonstrated that the water extract of parsley exhibited a low oxidation inhibitory effect using FRAP test, with an EC_50_ of 0.93 mmol/L [92,93]. Moreover, it has been proposed that a high TAC may be closely related to the presence of a high content of polyphenolics [94].

The antimicrobial activity of *P. sativum* Hoffm. polyphenolic fraction towards *S. aureus*, *P. aeruginosa*, and *C. albicans* highlights the potential use of this plant’s extract in treating bacterial and fungal wound infections. In fact, these pathogenic species along with others such as *S. epidermidis*, *Escherichia coli*, *Klebsiella pneumonia*, and *Proteus* are commonly isolated from infected wounds. Therefore, our findings provide useful information on the wound healing properties of plant-based chemicals through controlling infectious agents [95].

To explain the mechanisms of action of plant-derived antimicrobial compounds, several studies have attempted to correlate their antibacterial effect with their phytochemical composition [96]. Some scientists have suggested that the antibacterial action is associated with high concentrations of phenols, monoterpenes, aldehydes, and ketones that perturb the integrity of microbial membranes [97]. This may be due to the hydrophobic nature of some phytocompounds, allowing their accumulation on the cell membranes and disrupt their structure and function. This also weakens the microbial enzyme machinery, allows intracellular components to leak, and leads to apoptosis [98,99]. Other investigations have reported that these products can coagulate the microbial cytoplasm and bring down lipids and proteins [100]. Mostafa et al., (2020) recently reported that the stem bark extract from *Salix tetrasperma* impaired the virulence of *P. aeruginosa* by hindering its swimming and swarming on plates and by inhibiting its hemolytic and proteolytic activities [101]. Similarly, Ben bakrim et al., (2022) showed that the leaf extract from *Ximenia americana* var. *caffra* has the ability to inhibit the biofilm formation by the skin pathogen *P. aeruginosa* and reduced its mobilities in a dose-dependent manner [13]. Overall, the profile of a plant chemical composition may influence its targets, mechanisms of action, and consequently, its antibacterial activity. 

## 5. Conclusions

This study profiled the phytochemical composition of the hydro-ethanolic extract of *P. sativum* Hoffm. and highlighted some biological and pharmacological activities of its hydro-ethanolic extract and polyphenolic fraction. Our findings corroborate many previous investigations on the role of parsley-based phytochemicals such as polyphenols as analgesic, antioxidant, and antimicrobial agents. However, as far as we know, this is the first study to deliver proof that parsley could serve as a source of wound healing bioactive principles. Nevertheless, more evidence is needed to prove their direct influence using fractionation and guided bioassays and individual compounds isolation. In addition, further in-depth assessments should address the underpinning molecular and physiological mechanisms of observed analgesic and healing activities in vivo. Lastly, our study shed light on the potential and promising role of *P. sativum* Hoffm. as a source of analgesic, antimicrobial, and wound healing plant-based agents. 

## Figures and Tables

**Figure 6:**
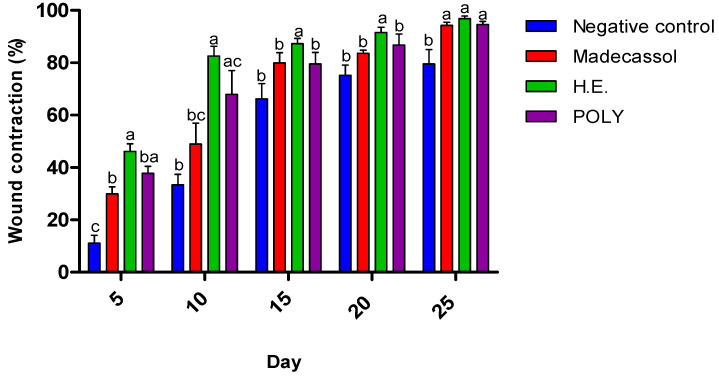
Burn healing activity of the hydro-ethanolic (H.E.) and polyphenolic (POLY) extracts of *P. sativum* Hoffm. and the control groups. Superscript letters (a, b, c) indicate the significant difference between treatments at *p* < 0.05.

**Table 1:** LC-MS/MS identification composition of *P. sativum* Hoffm. hydro-ethanolic extract.

Peak	Molecules	Formula	*R_t_* (min)	[M-H]- *(m/z)*	SI	Typical MS/MS	Area under Curve
1	Naringin	C_27_H_32_O_14_	1.108	579.00	579.2 → 124.89	271	27,397,987
2	Oleuropein	C_25_H_32_O_13_	1.115	539.00	539 → 179	307	56,757,543
3	Myricetin	C_15_H_10_O_8_	1.384	317.00	317 →150.8	287	3,417,929
4	Arbutin	C_12_H_16_O_7_	1.939	271.20	271.2 →144.9	162	28,442,471

**Table 2:** Identified compounds by GC-MS in the hydro-ethanolic extract of *P. sativum* Hoffm.

Peak	Name	Formula	*R_t_* (min)	Area %
1	l-Alanine, *N*-(trimethylsilyl)	C_9_H_23_NO_2_Si_2_	7.430	0.51
2	Cyclotetrasiloxane, octamethyl	C_8_H_24_O_4_Si_4_	8.148	1.60
3	Propanedioic acid, bis(trimethylsilyl)	C_9_H_20_O_4_Si_2_	8.417	1.08
4	L-Valine, *N*-trimethylsilyl	C_11_H_27_NO_2_Si_2_	8.558	0.72
5	Propanephosphonic acid, bis(trimethylsilyl)	C_9_H_25_O_3_PSi_2_	9.088	12.57
6	L-Isoleucine, *N*-(trimethylsilyl)	C_12_H_29_NO_2_Si_2_	9.248	0.62
7	Butanedioic acid, bis(trimethylsilyl)	C_10_H_22_O_4_Si_2_	9.317	0.75
8	L-Proline, 1-(trimethylsilyl)	C_11_H_25_NO_2_Si_2_	9.361	0.98
9	Benzonitrile	C_18_H_18_N_2_	9.594	0.62
10	Malic acid, tris(trimethylsilyl) ester	C_13_H_30_O_5_Si_3_	10.730	13.52
11	2,3,4-Trihydroxybutyric acid tetraTMS	C_16_H_40_O_5_Si_4_	11.260	0.56
12	D-Ribofuranose, 1,2,3,5-tetrakis-*O*-(trimethylsilyl)	C_17_H_42_O_5_Si_4_	12.918	5.62
13	β-D-Galactofuranoside, ethyl 2,3,5,6-tetrakis-*O*-(trimethylsilyl)	C_20_H_48_O_6_Si_4_	13.019	13.29
14	9*H*-Carbazole, 9-phenyl- alcaloide	C_18_H_13_N	13.220	3.04
15	Talose, 2,3,4,5,6-pentakis-*O*-(trimethylsilyl)	C_21_H_52_O_6_Si_5_	13.400	9.75
16	D-Mannitol, 1,2,3,4,5,6-hexakis-*O*-(trimethylsilyl)	C_24_H_62_O_6_Si_6_	13.653	10.95
17	D-Glucose, 2,3,4,5,6-pentakis-*O*-(trimethylsilyl)	C_21_H_52_O_6_Si_5_	13.782	13.52
18	D-gluconic acid 6TMS	C_24_H_60_O_7_Si_6_	14.071	1.31
19	Myo-Inositol, 1,2,3,4,5,6-hexakis-*O*-(trimethylsilyl)	C_24_H_60_O_6_Si_6_	14.549	1.19
20	Mannoonic acid, 2,3,5,6-tetrakis-*O*-(trimethylsilyl)	C_18_H_42_O_6_Si_4_	18.050	7.70
Total				99.9

**Table 3:** Antioxidant activity of hydro-ethanolic and polyphenols of *P. sativum* Hoffm. using DPPH and FRAP methods.

Extract	DPPH	FRAP
	IC_50_ (µg/mL)	EC_50_ (mg/mL)
Hydro-ethanolic extract	19.38 ± 0.15	5.34 ± 0.40
Polyphenols	40.36 ± 1.47	4.91 ± 0.40
BHT	01.97 ± 0.10	–
Ascorbic acid	–	1.43 ± 0.02

**Table 4:** Inhibition zone diameters of *P. sativum* Hoffm. extracts tested against bacterial and fungal species.

Inhibition Zone Diameter in mm			
Fractions	Gram-Negative Bacteria	Gram-Positive Bacteria	Fungi
	*P. aeruginosa*	*S. aureus*	*C. albicans*
Hydro-ethanolic extract	12.33 ± 0.33 ^a^	0.00 ± 0.00 ^b^	9.33 ± 0.33 ^ab^
Polyphenols	14.00 ± 0.33 ^a^	9 ± 0.16 ^a^	13 ± 0.57 ^a^
Streptomycin (1 mg/mL)	14.67 ± 0.17 ^a^	16 ± 0.57 ^a^	^__^
Fluconazole (5 mg/mL)	^__^	^__^	21 ± 1.2 ^a^

The different letters in superscript (a, b) indicate the significant difference between treatments at *p* < 0.05.

**Table 5:** MIC of *P. sativum* Hoffm. fractions tested against bacterial and fungal species.

Minimum Inhibitory Concentration (MIC) in mg/mL			
Fractions	Gram-Negative Bacteria	Gram-Positive Bacteria	Fungi
	*P. aeruginosa*	*S. aureus*	*C. albicans*
Hydro-ethanolic extract	12.5	Resistant	6.25
Polyphenols	6.25	3.125	6.25
Streptomycin	0.625	0.15	^__^
Fluconazole	^__^	^__^	0.31

**Table 6:** Effect of *P. sativum* Hoffm. on acetic acid-induced writhing in mice (*n* = 5).

Treatment	Dose (mg/kg)	Number of Writes
Control		65.00 ± 2.88 ^c^
Tramadol	10	17.33 ± 1.45 ^a^
Hydro-ethanolic extract	500	50.00 ± 0.57 ^bc^
	1000	39.67 ± 0.88 ^b^
Polyphenols	200	46.00 ± 3.05 ^b^

The different letters in superscript (a, b, c) indicate the significant difference between treatments at *p* < 0.05.

**Table 7:** Effect of the hydro-ethanolic and polyphenolic extracts of *P. sativum* Hoffm. on the response of mice upon the formalin-induced pain.

Treatment	Dose (mg/kg)	Licking Time (s)	
		First Phase (0–5 min)	Second Phase (15–30 min)
Control		58.00 ± 0.5 ^c^	30.33 ± 2.5 ^c^
Tramadol	10	10.33 ± 0.8 ^a^	4.33 ± 1.7 ^a^
Hydro-ethanolic extract	500	49.67 ± 1.4 ^b^	21.67 ± 2.1 ^b^
	1000	38.67 ± 1.7 ^b^	19.00 ± 1.1 ^b^
Polyphenols	200	43.00 ± 1.1 ^bc^	21.00 ± 0.5 ^b^

The different letters in superscript (a, b, c) indicate the significant difference between treatments at *p* < 0.05.

**Table 8:** Wound size (cm^2^) of the hydro-ethanolic and polyphenols extracts of *P. sativum* Hoffm. from day 1 till day 25.

Wound Size in cm^2^						
Treatments	Day 1	Day 5	Day 10	Day 15	Day 20	Day 25
Negative Control	1.91 ± 0.15 ^a^	1.70 ± 0.18 ^a^	1.26 ± 0.03 ^a^	0.62 ± 0.05 ^a^	0.46 ± 0.03 ^a^	0.37 ± 0.06 ^a^
Madecassol^®^ (1%)	2.27 ± 0.12 ^a^	1.59 ± 0.09 ^a^	1.14 ± 0.11 ^a^	0.46 ± 0.10 ^a^	0.37 ± 0.04 ^a^	0.13 ± 0.03 ^b^
Hydro-ethanolic extract (10%)	2.83 ± 0.03 ^a^	1.52 ± 0.06 ^a^	0.49 ± 0.10 ^b^	0.36 ± 0.05 ^a^	0.23 ± 0.05 ^a^	0.08 ± 0.02 ^b^
Polyphenolic extract	2.79 ± 0.46 ^a^	1.71 ± 0.23 ^a^	0.83 ± 0.15 ^ab^	0.58 ± 0.19 ^a^	0.36 ± 0.13 ^a^	0.14 ± 0.03 ^b^

The different letters in superscript (a and b) indicate the significant difference between treatments at *p* < 0.05.

## Data Availability

The data presented in this study are available in the main article and the supplementary materials.

## Supplementary Materials

The following supporting information can be downloaded at: https://www.mdpi.com/article/10.3390/metabo13020260/s1, Figure S1: XIC chromatograms of identified compounds.
